# One Problem, Many Solutions: Simple Statistical Approaches Help Unravel the Complexity of the Immune System in an Ecological Context

**DOI:** 10.1371/journal.pone.0018592

**Published:** 2011-04-19

**Authors:** Deborah M. Buehler, Maaike A. Versteegh, Kevin D. Matson, B. Irene Tieleman

**Affiliations:** 1 Department of Ecology and Evolutionary Biology, University of Toronto, Toronto, Ontario, Canada; 2 Department of Natural History, Royal Ontario Museum, Toronto, Ontario, Canada; 3 Animal Ecology Group, Centre for Ecological and Evolutionary Studies, University of Groningen, Groningen, The Netherlands; University of Utah, United States of America

## Abstract

The immune system is a complex collection of interrelated and overlapping solutions to the problem of disease. To deal with this complexity, researchers have devised multiple ways to measure immune function and to analyze the resulting data. In this way both organisms and researchers employ many tactics to solve a complex problem. One challenge facing ecological immunologists is the question of how these many dimensions of immune function can be synthesized to facilitate meaningful interpretations and conclusions. We tackle this challenge by employing and comparing several statistical methods, which we used to test assumptions about how multiple aspects of immune function are related at different organizational levels. We analyzed three distinct datasets that characterized 1) species, 2) subspecies, and 3) among- and within-individual level differences in the relationships among multiple immune indices. Specifically, we used common principal components analysis (CPCA) and two simpler approaches, pair-wise correlations and correlation circles. We also provide a simple example of how these techniques could be used to analyze data from multiple studies. Our findings lead to several general conclusions. First, relationships among indices of immune function may be consistent among some organizational groups (e.g. months over the annual cycle) but not others (e.g. species); therefore any assumption of consistency requires testing before further analyses. Second, simple statistical techniques used in conjunction with more complex multivariate methods give a clearer and more robust picture of immune function than using complex statistics alone. Moreover, these simpler approaches have potential for analyzing comparable data from multiple studies, especially as the field of ecological immunology moves towards greater methodological standardization.

## Introduction

### Background: One problem, many solutions

The immune system is a complex collection of interrelated and overlapping solutions to the problem of disease. In the vertebrate immune system these solutions include relatively general and constantly maintained (though variable) defences such as circulating leukocytes and antimicrobial proteins (constitutive innate immunity), general but induced responses such as fever and sickness behaviours (induced innate immunity), and more specific and induced responses such as the production of antibodies by B-cells (induced acquired immunity; [1]). Organisms employ these multiple mechanisms to prevent and limit the effects of disease. Immunological complexity, however, presents a problem for researchers interested in studying immune function in an ecological context because variation in immune function cannot be meaningfully captured using a single measure [2], [3], [4], [5]. Furthermore, incongruence is often found between different immune indices, even within a single study. For example, in house sparrows (*Passer domesticus*) phytohaemagglutinin (PHA) induced wing web swelling is positively correlated with survival, while specific antibodies to sheep red blood cell challenge (SRBC) are negatively correlated with survival ([6] and see [7] for a review of other examples). This incongruence hints at possible trade-offs within the immune system (see examples in [3]). Over the past few years, ecological immunologists have attempted to better understand immunological complexity by developing and using multiple methods to measure immune function and to analyze the resulting data. In a sense, the researchers are mirroring nature by applying multiple solutions to a complex problem. This approach has resulted in exciting progress, but standardizing assays to measure immune function and providing appropriate statistical tools to analyse these data have proven difficult.

To understand how ecological immunology achieved its current status, it is useful to review why ecologists study immunology and what led them to devise multiple ways to measure immune function. First, ecologists are interested in understanding natural variation in immune function and evaluating this variation from a cost-benefit perspective within an ecological and evolutionary context [8]. Second, because immune systems are so multidimensional, ecologists often want or need to quantify multiple aspects of immune function to fully answer a research question. Furthermore, for practical and philosophical reasons, ecologists are interested in which immune defences (if any) can be examined together as a single variable and which defences vary independently and must be measured separately [5]. Third, ecologists are interested in whether relationships among aspects of immune function are consistent within and generalizable among different organizational levels (e.g. from one individual, species, time point or environment to another). Some researchers suggest that selection or constraint may result in consistently correlated axes of immune defence (*sensu* [9], [10]), while others suggest that flexibility may be paramount so that specific types of pathogens can be targeted and different aspects of immune function can be used in different circumstances [3], [7]. In this paper, we explore the usefulness of several statistical approaches for addressing the second and third points above. These approaches test assumptions about how multiple aspects of immune function are related among different organizational levels.

The field of ecological immunology needs analytical tools that can simultaneously summarize variation in multiple measures of immune function. Few studies have empirically examined relationships among immune indices. Instead, ideas about immune system synergisms and trade-offs are often based on general immunological knowledge from humans, domesticated animals and other model systems (reviewed in [1], [3], [7], [11]) or rooted in life-history hypotheses (e.g. [9]). When undertaken, multivariate studies in ecological immunology often apply different statistical approaches and include different immune indices. For example, simple correlations have been used to examine relationships among aspects of immune function [12], [13] and among measures of immune function and antioxidants [14], [15]. Principle component analysis (PCA, see Table 1 for a summary of abbreviations) has been used to examine relationships among immune indices within an axis of immune function (e.g. constitutive immunity [5], [16], [17], [18]) and among multiple axes of immune function [19]. Thus, the field of ecological immunology lacks a statistical protocol for analysing and summarizing variation in multiple measures of immune function. This analytical gap is by no means unique to this field. Similar gaps have been effectively bridged, for example when relating multiple morphological traits (e.g. [20], [21]) and when relating multiple genotypic and phenotypic variables (e.g. [22]).

**Table 1:** Abbreviations used in the text, tables and figures.

**Statistical Tools**	
PCA	Principal components analysis
CPCA	Common principal components analysis
SEM	Structural equation modeling
**Waterfowl species**	
ALCG	Aleutian Canada goose
NABD	North American black duck
CHPT	Chilean pintail
SGPT	South Georgia pintail
LATE	Laysan teal
MUSC	Muscovy duck
NENE	Nene or Hawaiian goose
WWWD	White-winged wood duck
**Stonechat subspecies**	
KazXEur	Cross between Kazakh and European stonechats
EurXKen	Cross between European and Kenyan stonechats
**Immune indices**	
Het	Heterophil concentration
Eos	Eosinophil concentration
Lym	Lymphocyte concentration
Mon	Monocyte concentration
MCSa	Microbicidal capacity against *S. aureus*
MCCa	Microbicidal capacity against *C. albicans*
MCEc	Microbicidal capacity against *E.coli*
Lys	Complement mediated lysis
Agg	Natural antibody mediated agglutination
Hap	Haptoglobin-like activity

In this age of powerful statistical techniques, one possible solution to analyzing multiple datasets is the better and more consistent application of multivariate tools. For example, PCA and structural equation modelling (SEM; [23]) can be used to examine how immune indices are correlated within a single group, and common principal components analysis (CPCA; [24], [25]) can be used to examine whether indices correlate in the same way among multiple groups. However, in order to employ these methods across multiple studies, the same aspects of immune function need to be measured. Furthermore, if PCA or SEM are to be used with a single global dataset set (e.g. birds) that is composed of different groups (e.g. species), it must be assumed that relationships among immune indices are the same in the different groups. To our knowledge SEM has not yet been used in the context of ecological immunology, and although ecological immunologists have used PCA; the next step – using CPCA to test whether immune indices correlate in the same way among groups – has not yet been taken. We explore the usefulness of CPCA in this paper, and we illustrate the value of statistically simpler methods, such as pair-wise correlations, as complements to complex multivariate techniques. Although we do not use SEM, we end the paper with a brief discussion of its future potential for moving the field forward.

### Principal component analysis (PCA) and its assumptions

Principal component analysis is a method that derives linear combinations of the original variables in a dataset to summarize variation [26], [27]. PCA can be used to reduce many variables to fewer derived variables (principal components or PCs), which can then be used in further analyses. PCA can also be used to identify covariation among more than two variables. Ecological immunologists have used PCA to summarize data taken from multiple measures of immune function and to examine how the PCs vary over the annual cycle [16], between individuals or treatments [5], [16], [18], and among species [5], [19]. However, determining relationships among indices at these different organizational levels often requires pooling data from different groups (e.g. individuals in different months).

PCA provides a multivariate description of data structure within a single group, not among multiple groups [21]. Previously, this statistical issue has been dealt with by partitioning variation into different levels (i.e. among species and among individuals [5], or among individuals and over time [16]). For example, variation in indices of immune function has been described among units (i.e. individuals or months) belonging to multiple groups (i.e. species [5] or individuals [16]) by pooling across groups, in essence, ignoring group affiliation. However, this approach assumes that indices covary similarly among all groups that make up a global dataset. To our knowledge this assumption remains untested in the context of ecological immunology. Testing this assumption will provide new insight into immunological covariation. Consistent relationships may indicate physiological constraints, while labile relationships could have different interpretations depending on the organizational level (e.g. species-specific selection pressures, individual-specific immune strategies, season-specific responses).

### Testing this assumption

Several methods are available for testing this assumption. First, CPCA is a procedure that tests whether variables covary in a similar way in different groups. CPCA determines whether the variance-covariance matrices among groups are structurally similar, and the method differentiates among degrees of matrix similarity. Immune indices could covary in a similar way among groups (i.e. the matrices are equal). Immune indices could covary in a similar way, but with the strength of covariation differing among groups (i.e. the matrices are proportional). Some immune indices could covary in a similar way while others covary differently among groups (i.e. the matrices share some but not all PCs). Finally, the matrices could be completely unrelated [24], [25]. CPCA can serve as a powerful statistical tool, but several caveats should be taken into consideration when making biological interpretations based on CPCA [28]. CPCA, like PCA, assumes that relationships among the PCs are orthogonal. However, in ecology it is often unrealistic to assume that the underlying causes of the data structure are completely independent. For example, covariation in indices of immune function may be affected by hormone concentrations, which may in turn be correlated with one another. Furthermore, when CPCA determines that PCs are not common among groups, the analysis does not identify which or how particular relationships or variables are inconsistent [28].

In light of the limitations of CPCA, it is also useful to employ simple pair-wise correlations. To summarize correlations, we plot correlation coefficients using dot and boxplots. Plotting correlation coefficients individually allows for visual evaluation of whether particular groups are clustered or well-scattered. Plotting data as boxplots allows for a simple visual examination of how pairs of indices are related in general (the mean correlation) and whether correlations are consistent among groups or over time (the width of the boxes and whiskers).

**Figure 1:**
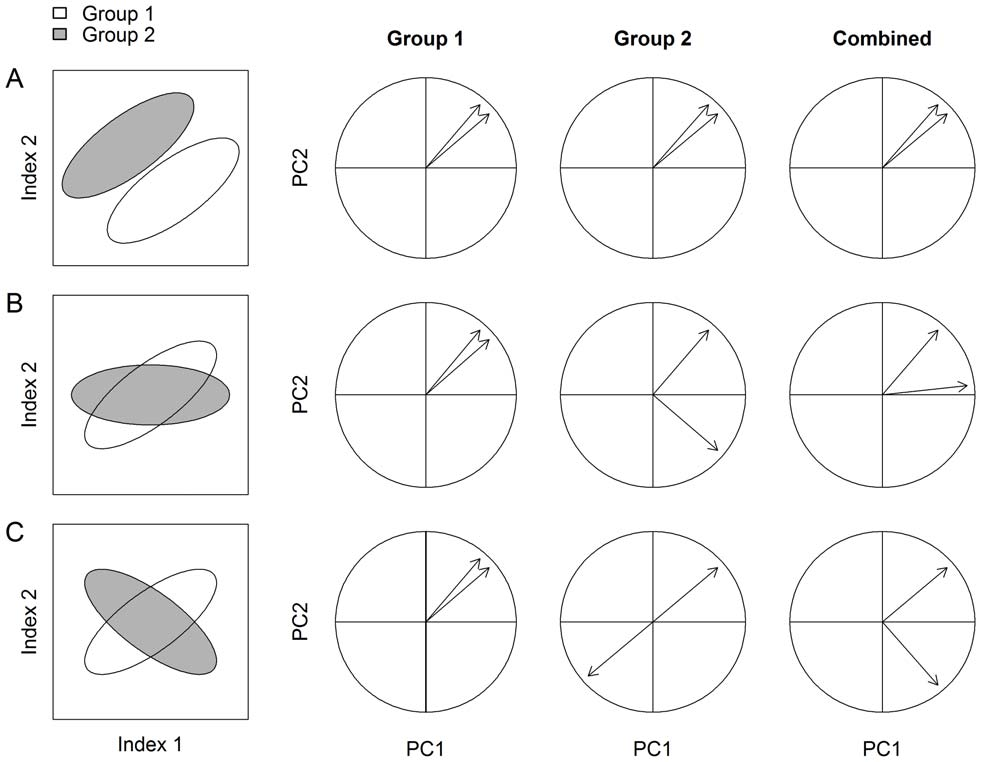
Simplified scenarios illustrating how correlation circles can be interpreted when relationships between variables among groups are similar (a), dissimilar (b), or opposite (c). The first vertical panel consists of a scatterplot showing the correlation between two indices in two groups (e.g. species, individuals or months). The next two panels show the resulting correlation circles for the two groups separately. The fourth panel shows the relationship given when the two groups are combined, and highlights how important patterns may be diluted (b), or missed (c). In the correlation circles, vectors are loadings for the two indices of immune function. The angle between two vectors gives the degree of correlation (adjacent = highly correlated, orthogonal (90°) = uncorrelated, and opposite (180°) = negatively correlated).

A final approach is to carry out separate PCAs and plot separate correlation circles for each group, for example species, subspecies, individual or time points. In a correlation circle, all of the original variables included in a PCA are plotted against two of the principal components, which are represented as the x- and y-axes. Using a simple, hypothetical dataset with only two immune indices and two groups, Figure 1 demonstrates how these diagrams can be interpreted. In (a) the relationships between the variables are similar in the two groups, in (b) they differ somewhat, and in (c) they are opposite. The fourth panel of Figure 1 highlights how important patterns may be diluted (b) or missed (c) when groups are pooled. Correlation circles allow for graphical examination of the relationships among indices, and the consistency of these relationships among groups and over time. This approach can supplement the pair-wise correlation approach. While that approach allows simultaneous evaluation of bivariate relationships, the separate PCAs and correlation circles allow for simultaneous examination of covariation among multiple immune indices.

We used these three methods to test the assumption of consistent relationships among immune indices at the species, subspecies, individual and temporal levels. We analysed one unpublished and two previously published multivariate datasets of immune function: (1) multiple species of waterfowl at a single time point [5], (2) multiple subspecies of a passerine [Versteegh et al. unpublished data] during a single season and (3) multiple individuals of a single shorebird species over the annual cycle [16].

## Methods

### Datasets

Matson et al. [5] measured 13 indices of immune function in 10 species of waterfowl using five general protocols: microbicidal activity, leukocyte concentrations, hemolysis-hemagglutination titers, haptoglobin-like concentration, and antioxidant capacity. In Matson et al. [5] the data were split into plasma-based and cellular indices, because sample sizes within each species did not allow for PCA of all of the variables in a single analysis. In this study we restricted our analyses to five indices immune function derived from plasma samples: microbicidal capacity of plasma against *Escherichia coli* and *Staphylococcus aureus* (anti*-E.coli* and anti*-S. aureus* capacities*)*; hemolysis and hemagglutination titers against rabbit red blood cells; and haptoglobin-like concentration (see Table 1 for abbreviations). We omitted hemolysis and hemagglutination titers against trout blood since most researchers use only rabbit blood and because trout and rabbit blood gave very similar results [5]. We omitted antioxidant capacity in order to limit the examined indices to those with a primarily immunological function. We excluded two of the ten species because sample sizes (n = 2 and n = 1) were not large enough to run within-species PCA. Therefore, the final dataset included eight species: Aleutian Canada goose, *Branta canadensis leucopareica* (ALCG, n = 7); North American black duck, *Anas rubripes* (NABD, n = 6); Chilean pintail, *Anas georgica spinicauda* (CHPT, n = 8); South Georgia pintail, *Anas georgica georgica* (SGPT, n = 8); Laysan teal, *Anas laysanensis* (LATE, n = 8); Muscovy duck, *Cairina moschata* (MUSC, n = 5); nene or Hawaiian goose, *Branta sandvicensis* (NENE, n = 8); white-winged wood duck, *Cairina scutulata* (WWWD, n = 8, Table 1). Although we also analysed the cellular indices of immune function, these results are presented in the supplementary material only.

Versteegh et al. (unpublished) measured six indices of immune function in four subspecies and two hybrid populations of captive stonechats, using three general protocols: microbial capacity against *E. coli*, *S. aureus* and *Candida albicans*, hemolysis-hemagglutination titers and haptoglobin-like activity. All individuals were measured during a single quiescent period in the annual cycle (winter). The geographically-distinct subspecies originated from Kazakhstan (*Saxicola torquata maura*, n = 10), Europe (*S. t. rubicola*, n = 15), Kenya (*S. t. axillaris*, n = 13) and Ireland (*S. t. hibernans*, n = 16). The hybrids were crosses between Kazakh and European stonechats (KazXEur, n = 12) and European and Kenyan stonechats (EurXKen, n = 20, Table 1). Hybrid groups are henceforth referred to as subspecies for simplicity.

Buehler et al. [16] measured 14 indices of immune function in red knots (*Calidris canutus*) using three general protocols: microbicidal capacity, leukocyte concentrations and hemolysis-hemagglutination titers. We restricted our analyses to the 27 birds measured over the entire study period of 11 consecutive months. We also restricted our analyses to eight indices of immune function: a single time point in three strains for microbicidal capacities (*E. coli* after 10 min, *C. albicans* after 60 min and *S. aureus* after 120 min); concentrations of heterophils, lymphocytes and monocytes; and hemolysis and hemagglutination titers against rabbit red blood cells. Total leukocyte concentrations were excluded since they are the sum of the differential concentrations; eosinophil concentrations were excluded due to a large number of 0 values; thrombocyte concentrations were excluded because these cells are not commonly quantified by ecologists. We used the transformed leukocyte concentration data to ensure normality and to maintain consistency with Buehler et al. [16].

Anti-*E. coli* and anti-*S. aureus* capacities were measured in all three datasets, but the results are not entirely comparable. Matson et al. [5] used plasma, which indicates the microbicidal capacity of soluble blood components alone. Buehler et al. [16] and Versteegh et al. (unpublished) used whole blood, which potentially reflects actions by both cellular (e.g. phagocytosis) and soluble components.

### Statistical methods

#### Common principal component analysis (CPCA): A statistical test of matrix similarity

Common principal component analysis is an extension of PCA that compares the structure of two or more covariance matrices in a hierarchical fashion. This hierarchy is built on the recognition that any two matrices can relate to one another in a complex fashion and that the two are not simply equal or unequal. CPCA determines whether matrices are equal, proportional, share a number of principal components, or are unrelated. The number of principal components shared can range from one to *p*-2, where *p* is the number of variables. CPCA relies on two approaches — the model-building approach and the step-up approach — to identify the most likely relationships among matrices. In the model building approach, the best model is the model with the lowest Akaike information criterion (AIC). In the step-up approach, a null-hypothesis is iteratively compared to an alternative hypothesis. The compared models are chosen from a hierarchy, which begins with two unrelated matrices, and progresses through matrices that share one PC, that share more than one (up to *p*-2) PC, that are proportional, and, finally, that are equal. The null model is always tested against an alternative model one step higher in the hierarchy. A significant P-value means that the null model better describes the data [25].

We used the program CPC [29], which performs the analysis described by Flury [24], to carry out CPCA. We used correlation matrices rather than covariance matrices because we were interested in the relationship between indices independent of their absolute values. We calculated correlation matrices for each species, subspecies, individual and month. For the comparisons among individuals and among months, the same data were used, but the correlation matrices were constructed per bird or per month respectively. The CPCA of plasma indices for waterfowl species was based on seven matrices with six to eight observations per matrix (the MUSC species was excluded due to low levels of variability in one or more immune index); the CPCA of stonechat subspecies was based on six matrices with 12 to 20 observations each; the CPCA of individual red knots was based on 27 matrices with 11 observations each; and the CPCA of red knots over time was based on 11 matrices with 27 observations each.

#### Pair-wise correlations and boxplots

We calculated Pearson correlation coefficients for all pairs of immune indices. The correlation coefficients are presented as boxplots for each pair of indices at a particular level of analysis (i.e. species, subspecies, individual and months). For example, at the species level, each box with whiskers represents the distribution of eight species-specific correlations and each correlation is based on 5 to 8 individuals. Furthermore, we calculated 95% confidence intervals and performed t-tests to examine whether the mean correlation coefficients for each pair-wise comparison differed significantly from zero (after accounting for multiple comparisons using a sequential Bonferroni correction [30]). Because correlation coefficients are not normally distributed, we transformed the correlations into z-scores ([31] page 578) before carrying out the t-tests. For waterfowl and stonechats, sample sizes differed among species or subspecies. Therefore, we calculated weights based on sample sizes in such a way that the group (or groups) with the largest sample size were given a weight of 1 (adapted from [31] page 42). We multiplied the z-transformed correlation coefficients with these weights to obtain weighted correlations. Using these weighted correlations, we calculated means and variances and performed t-tests (n =  the number of groups in the analysis). T-tests performed on weighted and unweighted correlations resulted in similar outcomes. The boxplots in combination with these t-tests allowed us to gauge both the consistency of the correlations (width of a box/whiskers) and whether or not mean correlations differed significantly from zero. Finally, correlation coefficients were plotted individually in dotplots and coded by species, subspecies, or “subpopulation” (i.e. treatment group or month in the case of knots [16]). These dotplots were examined for clustering of points, which allowed us to explore the possibility of consistent correlations in one or more subsets. For example, a mean correlation coefficient near zero and a wide box and whiskers could be the result of two indices being positively correlated in some groups and negatively correlated (or consistently uncorrelated) in other groups. Pearson correlations, plots and t-tests were performed using R [32].

#### Within-group principal component analysis (PCA) and correlation circles

We carried out separate PCAs and plotted a separate correlation circle for each group at each organizational level (species, subspecies, individual or time point), to concurrently examine the relationships among indices and among groups. This analysis allows the examination of multiple dimensions at once (i.e. immune indices), but one potential limitation is that all indices must be measured in all groups.

PCAs were performed and correlation circles were generated using the ade4 package in R [33]. In every correlation circle, each measured variable is shown as a vector, which signals the combined strength of the relationships between the measured variable and two PCs (vector length) and whether these relationships are positive or negative (vector direction). The angle between two vectors signals the degree of correlation between two measured variables. A right angle indicates that two variables are completely uncorrelated; zero or 180 degrees between two variables indicates complete positive or negative correlation. Consistent relationships among groups at a particular level (i.e. species, individual, month) are indicated by similar angles among the vectors in different correlation circles. While we only show correlation circles with PC1 and PC2, we examined correlation circles for all combinations of PCs 1 to 4 (not shown). Unless otherwise stated, all correlation circles resulted in the same conclusions as those showing PC1 and PC2 alone.

We based the PCAs on the same correlation matrices used for the CPCA. Unlike Matson et al. [5] and Buehler et al. [16], we chose not to do a varimax rotation because we wanted to facilitate comparison between the PCAs and the CPCAs (which operate on unrotated values). Nevertheless, we shaded immune index labels in the group-specific correlation circles to reflect the PCs resulting from the previous varimax-rotated PCA performed with all species, individuals or months combined. Although this graphical representation is not completely comparable because rotation can affect PC composition, angles between vectors indicate the relationship between the variables in both rotated and unrotated PCAs. Therefore, if the previous analysis and the current analysis are in agreement, then indices having the same shading should be clustered together (if positively correlated) or in opposition (if negatively correlated), at least in the majority of cases.

## Results

### Common principal component analysis (CPCA)

Among waterfowl species, both CPCA approaches indicated complete shared structure with differing eigenvalues for both for plasma (CPC; Table 2) and cellular components (CPC; Table S1). Among stonechat subspecies, the two approaches indicated slightly different structures for plasma components. The step-up approach suggested complete shared structure with differing eigenvalues (CPC), and the model building approach suggested equality among matrices (Table 3). Among red knot individuals, the two approaches to CPCA resulted in the most extreme possible contradiction. The step-up approach suggested that the matrices were unrelated, whereas the model building approach suggested that the matrices were equal (Table 4). Finally, among months in individual red knots, the step-up approach indicated that the matrices shared all PCs but had different eigenvalues (CPC), and the model-building approach indicated that the matrices were equal (Table 5).

**Table 2:** Common principal components analysis (CPCA) of covariance matrices among waterfowl species for plasma-based indices of immune function.

Hierarchy					
Higher	Lower	χ2	df	*P*	AIC
Equality	Proport	0.570	6	0.9969	126.0
Proport	**CPC**	62.021	24	**<0.0001**	137.4
**CPC**	CPC(3)	4.524	6	0.6062	**123.4**
CPC(3)	CPC(2)	19.496	12	0.0772	130.9
CPC(2)	CPC(1)	15.393	18	0.6348	135.4
CPC(1)	Unrelated	23.977	24	0.4629	156.0
Unrelated	—				180.0

The table shows Flury's Decomposition of Chi Square using step-up and model building approaches [24], [25]. In the step-up approach at each step in the hierarchy the hypothesis labeled “higher” is tested against the hypothesis on the step below, “lower”. The hierarchy is built in a step-wise fashion starting with no relation between the matrices (Unrelated) and rising to CPC(1), then CPC(2), etc, through CPC, Proportionality, and Equality. The likelihood that a particular model is valid is given by the *P*-value, thus low *P*-values indicate a low probability that the higher model is better than the lower model [25]. The best solution can also be evaluated using the model building approach where the best model is indicated as the “higher” model in the row with the lowest Akaike information criterion (AIC). Both methods indicate that the matrices share all PCs in common but have different eignevalues (CPC).

**Table 3:** Common principal components analysis (CPCA) of covariance matrices among stonechat subspecies.

Hierarchy					
Higher	Lower	χ2	df	*P*	AIC
**Equality**	Proport	1.015	5	0.9614	**103.7**
Proport	**CPC**	47.044	25	**0.0048**	112.7
**CPC**	CPC(4)	8.443	5	0.1335	115.6
CPC(4)	CPC(3)	4.514	10	0.9212	117.2
CPC(3)	CPC(2)	10.241	15	0.8043	132.7
CPC(2)	CPC(1)	13.959	20	0.8326	152.4
CPC(1)	Unrelated	18.464	25	0.8221	178.5
Unrelated	—				210.0

The step-up approach (where a low p-value indicates a low probability that the higher model is better than the lower model) suggests that covariance matrices among subspecies share all PCs, but have differing eigenvalues (CPC), and the model building approach (where the lowest AIC value indicates the best model) suggests that the matrices are equal (see Table 1 for a complete description of the step-up and model building approaches).

**Table 4:** Common principal components analysis (CPCA) of covariance matrices containing indices of immune function among individual red knots.

Hierarchy					
Higher	Lower	χ2	df	*P*	AIC
**Equality**	Proport	6.978	26	0.9999	**1464.2**
Proport	CPC	281.317	182	<0.0001	1509.3
CPC	CPC(6)	33.538	26	0.1471	1591.9
CPC(6)	CPC(5)	64.881	52	0.1083	1610.4
CPC(5)	CPC(4)	88.281	78	0.1998	1649.5
CPC(4)	CPC(3)	152.434	104	0.0014	1717.2
CPC(3)	CPC(2)	214.075	130	<0.0001	1772.8
CPC(2)	CPC(1)	304.889	156	<0.0001	1818.7
CPC(1)	**Unrelated**	317.835	182	**<0.0001**	1825.8
**Unrelated**	—				1872.0

**Table 5:** Common principal components analysis (CPCA) of covariance matrices containing indices of immune function among 11 month within individual red knots.

Hierarchy					
Higher	Lower	χ2	df	*P*	AIC
**Equality**	Proport	2.189	10	0.9947	**362.8**
Proport	**CPC**	122.267	70	**0.0001**	380.6
**CPC**	CPC(6)	17.194	10	0.0702	398.3
CPC(6)	CPC(5)	22.560	20	0.3109	401.1
CPC(5)	CPC(4)	18.012	30	0.9583	418.5
CPC(4)	CPC(3)	29.036	40	0.9004	460.5
CPC(3)	CPC(2)	50.667	50	0.4471	511.5
CPC(2)	CPC(1)	47.340	60	0.8823	560.8
CPC(1)	Unrelated	53.495	70	0.9286	633.5
Unrelated	—				720.0

### Pair-wise correlations and boxplots

**Figure 2:**
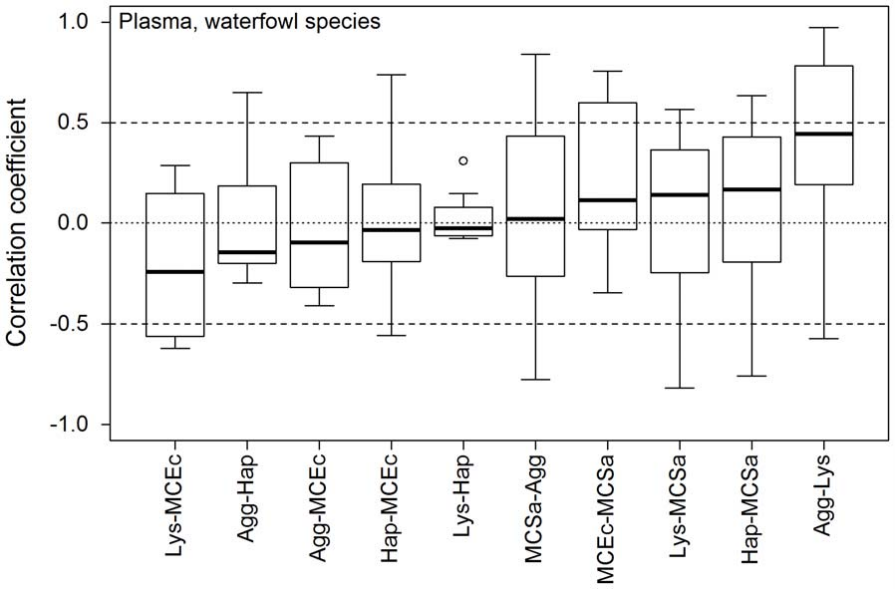
Boxplots showing correlation coefficients for pair-wise Pearson correlations between plasma-based indices of immune function among waterfowl species (n = 8, see Table 1 for abbreviations). Solid lines indicate the median, boxes the inter-quartile range, whiskers the range and open circles outliers. Lower variation (smaller boxes, whiskers and few outliers) indicates more consistent correlations among species.

**Figure 3:**
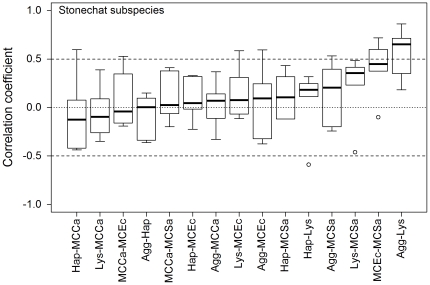
Boxplots showing correlation coefficients for pair-wise Pearson correlations between indices of immune function among stonechat (*Saxicola torquata*) subspecies (n = 12 to 20, see methods for sample size details and Table 1 for abbreviations). Solid lines indicate the median, boxes the inter-quartile range, whiskers the range and open circles outliers. Lower variation (smaller boxes, whiskers and few outliers) indicates more consistent correlations among subspecies. Asterisks indicate weighted mean correlation coefficients [31] that differed significantly from zero after sequential Bonferroni correction [30] (see Tables S3 for statistics).

Box and whisker plots of waterfowl species-specific correlation coefficients were wide (median IQR = 0.47), box widths varied (0.14<IQR<0.71), and the narrowest box exhibited an outlier (Figure 2; see Table 1 for immune index abbreviations). Furthermore, 95% confidence intervals and t-tests indicated that none of the mean correlation coefficients differed significantly from zero (Table S2). Combined, these results suggest that in this dataset pairs of immune indices are weakly and inconsistently correlated among waterfowl species. Pair-wise correlations among stonechat subspecies showed slightly less variability than among waterfowl species (0.12<IQR<0.49, median IQR = 0.32; Figure 3), but three boxes had outliers. Furthermore, only one of the 15 mean correlation coefficients differed significantly from zero (Agg-Lys; Table S3).

**Figure 4:**
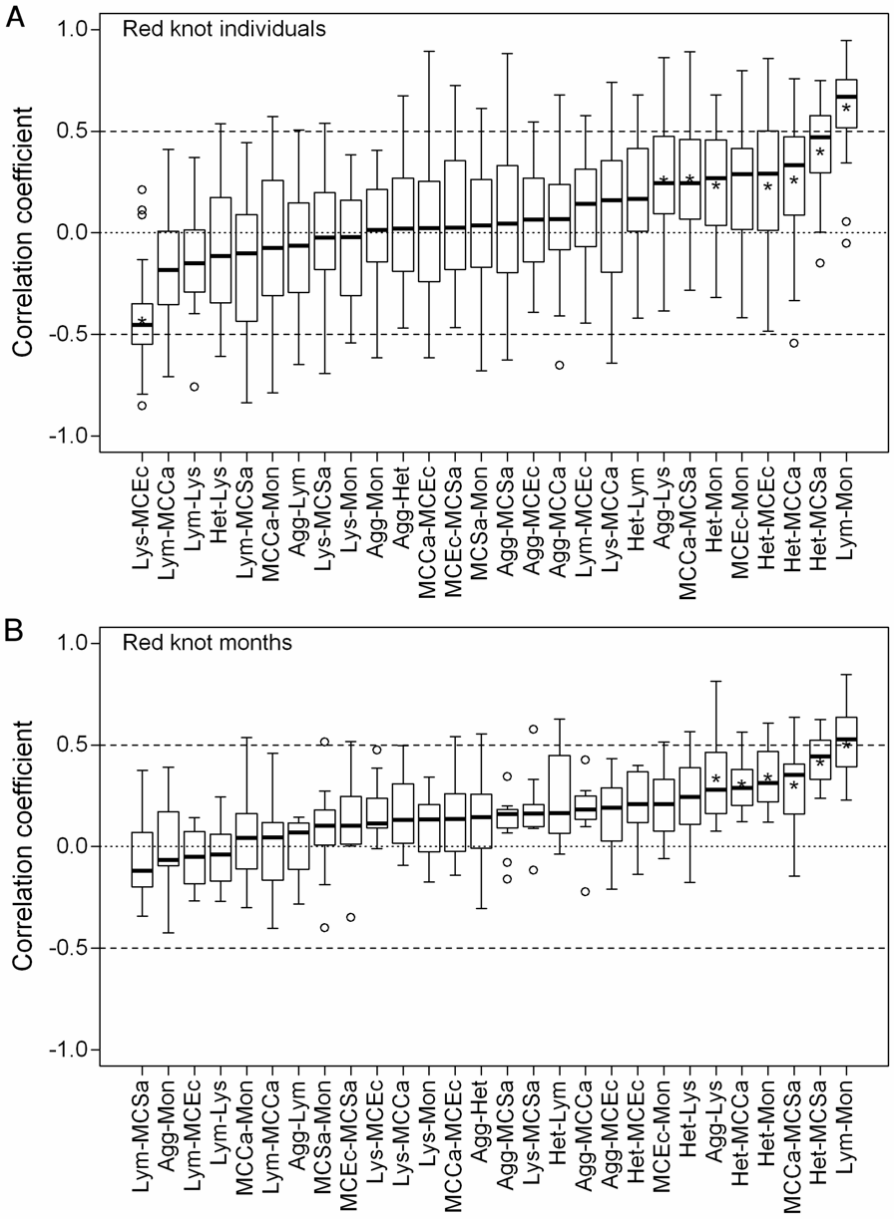
Boxplots showing correlation coefficients for pair-wise Pearson correlations between indices of immune function (see Table 1 for abbreviations) in red knots (*Calidris canutus*) among individuals (a, n = 27 birds), and over time (b, n = 11 monthly measurements). Solid lines indicate the median, boxes the interquartile range, whiskers the range and open circles outliers. Lower variation (smaller boxes, whiskers and few outliers) indicates more consistent correlations. Asterisks indicate mean correlation coefficients that differed significantly from zero after sequential Bonferroni correction [30] (see Tables S4 for statistics).

Pair-wise correlations among knot individuals showed a similar degree of variability as was seen among species and subspecies (0.20<IQR<0.61, median IQR = 0.41; Figure 4a). The most positively (lymphocytes-monocytes) and negatively (hemolysis-anti-*E.coli* capacity) correlated pairs had whiskers that did not include zero, but even these pairs had outliers that crossed zero. However, eight of the 28 relationships had mean correlation coefficients that differed significantly from zero (Figure 4a and Table S4a). Within individual knots over 11 months, box and whisker plots were narrower than at any other level (0.09<IQR<0.55, median IQR = 0.25; Figure 4b). Furthermore, 6 of the 28 relationships were significantly different from zero (Table S4b) and of the 6 significant pairs, only one had whiskers that included zero and none had outliers. Thus, some indices were consistently correlated, either positively or negatively, over the year.

Dotplots of individual correlation coefficients (not shown) indicated no clustering among subsets of waterfowl species or stonechat subspecies. Furthermore, in red knots we found no clustering by treatment (i.e. warm, cold, and variable) or by season (migration, moult, wintering).

### Within-group principal component analysis (PCA) and correlation circles

**Figure 5:**
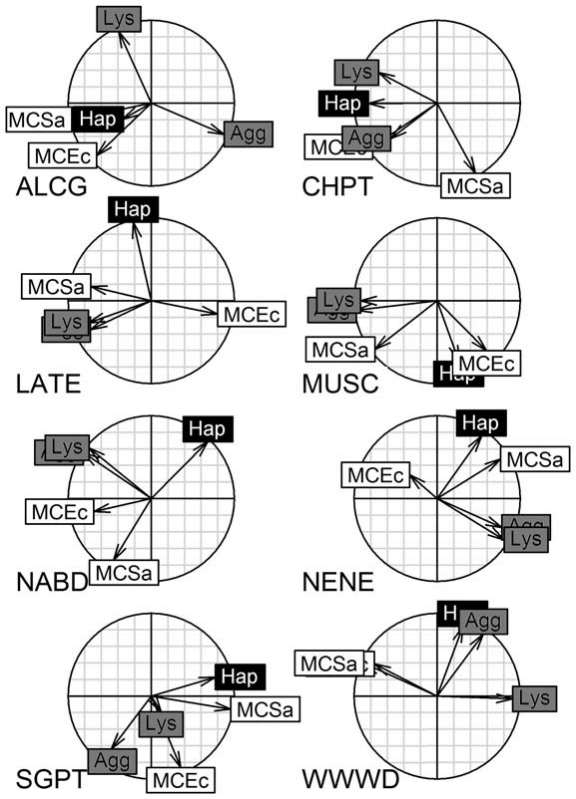
Correlation circles for unrotated principal component analyses (PCA) on plasma-based immune indices among waterfowl species (see Table 1 for abbreviations). Vectors are the loadings on PC1 (x-axis) and PC2 (y-axis). Vector length indicates the strength of the relationship and the angle between two vectors gives the degree of correlation (adjacent = highly correlated, orthogonal (90°) = uncorrelated, and opposite (180°) = negatively correlated). Shading indicates how the indices of immune function were grouped in a previous varimax rotated PCA performed with all species combined [5]. Indices having the same shading were associated with the same PC in the previous analysis.

The angles between vectors in the species-specific correlation circles differed widely indicating that relationships among plasma (Figure 5) and cellular (Figure S1) indices were generally inconsistent among waterfowl species. For example, anti-*E.coli* capacity and hemagglutination were uncorrelated (perpendicular) in ALCG and highly correlated (parallel) in CHPT. An exception is the uncorrelated relationship (perpendicular) between haptoglobin and hemolysis, which was consistent among species (also reflected by a narrow box in Figure 2). Shaded variable labels in Figure 5 indicated that immune indices that were related in the previous analysis [5] were not related when the PCA was performed on each species separately.

**Figure 6:**
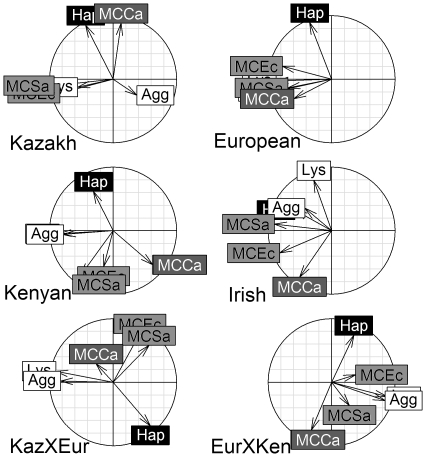
Correlation circles for unrotated principal component analyses (PCA) on immune indices among stonechat subspecies (see Table 1 for abbreviations). Vectors are the loadings on PC1 (x-axis) and PC2 (y-axis). Vector length indicates the strength of the relationship and the angle between two vectors gives the degree of correlation (adjacent = highly correlated, orthogonal (90°) = uncorrelated, and opposite (180°) = negatively correlated). Shading indicates how the indices of immune function were grouped in a varimax rotated PCA performed with all subspecies combined (see Table S5). Indices having the same shading were associated with the same PC in the combined PCA. Anti-*C. albicans* capacity correlated equally across two PCs in the pooled analysis (Table S5), therefore it has darker grey shading with white lettering.

Subspecies-specific correlation circles indicated that some immune indices were consistently related among stonechat subspecies; other indices were consistently related in some, but not all subspecies; and yet other indices were not consistently related among subspecies (Figure 6). For example, hemolysis and hemagglutination were positively correlated in all subspecies. The angles between the vectors of these indices were small in five of the six subspecies (Figure 6); and in Kazakh birds, although the angle was perpendicular the vectors were short, indicating that they were not strongly correlated with PC1 or PC2. Examination of the first four PCs showed that they were positively correlated with a high loading on the third PC (not shown). Anti-*E. coli* and anti-*S. aureus* were positively correlated in some, but not all subspecies. The angles between the vectors of these indices were small in five of the six subspecies (Figure 6). In EurXKaz subspecies the angle was perpendicular and the vectors were short. Examination of the first four principal components showed that these two indices were negatively correlated on the third PC (not shown). Finally, haptoglobin and hemagglutination showed completely different relationships in the different subspecies. They were positively related in Irish birds, unrelated in EurXKen hybrids and negatively related in Kazakh birds. Shaded variable labels (Figure 6) indicated that immune indices that were related in a varimax rotated PCA combining all subspecies (Table S5, see [5] for method) clustered in some (e.g. European) but not all (e.g. KazXEur) subspecies when the PCA was performed on each subspecies separately.

Individual-specific correlation circles indicated that relationships among some indices were consistent among most red knot individuals (Figure S2a). However, not all indices were consistently related in all individuals. For example, the angles between monocytes and lymphocytes are small in most, but not all birds (see birds 19 and 24, Figure S2a). In this way, the correlation circle results supported boxplot results. Immune index pairs with narrow boxes (Figure 4) are the same indices that group together in most correlation circles (e.g. lymphocytes and monocytes). Shaded variable labels in Figure S2a indicated that immune indices that grouped together previously when all birds were combined in a single analysis [16], once again cluster in most birds (e.g. lymphocytes and monocytes), but exceptions remain (see birds 19 and 24).

Finally, month-specific correlation circles indicated that relationships among indices were relatively consistent through the year (Figure S2b). For example, the angle between lymphocytes and monocytes is small in all months. Shaded variable labels showed that immune indices that grouped together previously [16] again clustered fairly consistently when all months were analyzed separately (i.e. lymphocytes, monocytes; heterophils and anti-*C. albicans* and anti-*S. aureus* capacities, Figure S2b).

## Discussion

To deal with the complexity of the immune system, ecological immunologists have devised multiple techniques to measure different aspects of immune function in wild animals. In this paper we address the question of how multivariate immunological datasets can be meaningfully analyzed and interpreted among distinct groups at a particular organizational level (e.g. species or populations). Previously, researchers have applied multivariate methods to data that are pooled after statistically neutralizing any group-effect. However, the implicit assumption is that relationships among the assayed immune indices are similar in all groups. We tested this assumption using CPCA, and then employed pair-wise correlations and correlation circles to delve deeper into the consistency and inconsistency of these relationships among groups. These statistical analyses range in complexity and in novelty, at least in terms of their use with multivariate datasets concerning immune function. Nonetheless, when packaged together and treated as a statistical protocol, these methods can serve as an important new tool, which can be applied to a variety of datasets and organizational levels.

### Fresh insights provided by this novel statistical approach

The current statistical protocol gave insight that was inaccessible when using previous analytical approaches. As a result, the presented protocol facilitates more-detailed conclusions than were previously possible (e.g. [5] [16] [19]) and, thus, acts as a foundation for new hypotheses. We highlight these new insights by comparing and contrasting current and prior results at different organization levels.

Overall, the extent to which multivariate patterns are consistent among groups at different organizational levels varies. For example, relationships among immune indices differed from species to species, but relationships among immune indices were fairly similar from month to month. This variability could depend on qualities of the dataset, the organizational level, or both. Once additional studies begin employing this statistical protocol, a picture will emerge as to whether any generalities about specific organizational levels can be made. For instance, patterns among immune defenses might typically be similar among populations but different among species. In fact, our results suggest that relationships among immune indices become less consistent as the complexity of the organizational level increases (i.e. from individuals to subspecies to species). If this pattern holds as additional datasets and analyses come to light, then it could serve as a basis for new hypotheses. For example, over shorter timescales physiological mechanisms might limit plasticity and variability in how immune defences relate to one another, but over longer time scales selection pressures might gradually reshape physiology and lead to decoupled and reorganized immunological relationships [34].

The current protocol allowed for the re-examination and refinement of previous multivariate analyses of immune function. Matson et al. [5] pooled individuals across species and reported a general among-individual pattern, which parallels assay category. The current approach, however, revealed that this use of pooling individuals might unduly influence the results of the analysis, since among-individual patterns differed by species. Buehler et al. [16] pooled their data in two ways: 1) data points from a given individual were pooled by statistically correcting for month to examine among-individual patterns 2) data points from a given month were statistically corrected for individual to examine among-month patterns. Our current approach suggested that the suitability of pooling differed between these two cases. On the one hand, statistically correcting for month revealed among individual patterns that appear to be well-supported since we demonstrate that relationships among immune indices were consistent from one month to the next. On the other hand, statistically correcting for individual to examine annual patterns might inappropriately shape the global relationships among immune indices since we demonstrate that these relationships varied from individual to individual. To summarize, if correlations are consistent among groups of an organizational level, then both our novel approach and the approach of pooling data across groups give similar results. However, if correlations are inconsistent across among groups of an organizational level, then a single combined analysis can obscure patterns.

Differences between the two approaches also hinge on issues of inference space and sample size. The general patterns derived from the analyses of pooled study subjects can potentially offer greater inference (e.g. the order Anseriformes as opposed to the species *Anas platyrhynchos*). One caveat is that in pooled analyses disparities in sample sizes among groups can affect patterns: a well-represented group with more measurements will exert greater influence on the global pattern than a poorly represented group with few measurements. While the groups we compared were generally similar in sample size, checking the consistency of correlations among groups is particularly important when sample sizes vary in such a way that one or a few groups dominate over many others. In the latter case, any evaluation of global patterns should be preceded by group-by-group evaluations. Another caveat is that the value of a general pattern based on a pooled analysis is dictated by the diversity of the representative groups. Ultimately, this too is an issue of sample size, albeit one concerning the number of groups, as opposed to the number of measurements per group.

### Immunological consistency and inconsistency among groups

The statistical protocol outlined in this paper provided insights into consistencies and inconsistencies in the way that immunological indices interrelate among groups. The usefulness of the protocol, however, extends beyond these particular datasets. The currently-analyzed organizational levels are meant to exemplify the applicability of this protocol to other organizational levels and possibly even to other multivariate datasets unrelated to immune function. Nevertheless, to provide a robust foundation for the future use of this protocol, we discuss briefly the results at each analyzed level.

Among waterfowl species, the CPCA indicated complete common principal component structure. This result suggests that covariance matrices are not statistically different among species. That is, immune indices interrelate similarly among species, and therefore pooling individuals into a single PCA is valid [5]. On the contrary, pair-wise correlation boxplots and species-specific correlation circles provided no evidence for consistent immunological relationships among species. The conflicting results might stem from small within-species sample sizes (n = 6–8 individuals), which can increase the likelihood of recovering shared covariance structure with CPCA even when little similarity actually exists [35]. Consequently, we cannot unequivocally conclude that immunological relationships are consistent within the tested species, and, the results seemingly invalidate the approach of pooling individuals of different species into a single PCA.

Among stonechat subspecies, the CPCA indicated either equality or complete common PC structure. That is, immunological relationships are consistent among subspecies. However, pair-wise correlation boxplots and subspecies-specific correlation circles cast some doubt on this result. While some immune indices were consistently related among subspecies, others were not. Conclusions similar to those at the species level can be drawn. Although the CPCA indicated shared PCs, the other methods cautioned against pooling all individuals across subspecies into a single PCA analysis.

Among individual red knots, the two CPCA approaches gave highly divergent results. The step-up approach indicated that relationships among immune indices differed among individuals; the model-building method suggested equality. The pair-wise correlation boxplots and the correlation circles showed that some immune indices are positively or negatively correlated in some, but not all, individuals. These inconsistent relationships are documented by large amounts of variation (i.e. wide boxes and whiskers) in the boxplots and by different angles between vector pairs in the array of correlation circles. These inconsistent relationships, apparent upon graphical examination, seemed to influence the step-up approach, and they may explain why this approach resolved that the matrices were different. Overall, the concurrence between the graphical approaches and the CPCA step-up approach suggested that immunological relationships were inconsistent at the individual level.

Among months, the two CPCA approaches and the two graphical approaches suggested that immunological relationships were consistent. Furthermore, immunological relationships that were previously identified via PCA on data pooled among months [16] were similarly identified in individual months. These results indicate that although absolute levels of immune indices are flexible over the annual cycle [16], relationships among these indices are relatively consistent. For example, index A can be high on average in one month and low on average in another, but within each month, indices A and B always correlate without regard to absolute levels.

### Future analyses: opportunities and challenges

The datasets we analyzed focused on indices of constitutive levels of innate immunity. Conducting similar analyses on datasets that include additional immune indices will be instructive. For example, Martin et al. [19] present a dataset that includes indices of constitutive innate immunity, acquired immunity, and induced responses. Potentially, this array of indices could be useful for examining whether relationships among types of immune defences, which represent putative “arms” of the immune system, are consistent among species. However, application of the protocol presented here requires separate species-specific PCAs, so all immune indices must be measured in the same individuals within each species. Unfortunately in Martin's dataset different individuals were used [19].

Our analyses demonstrated the value of graphical examination of pair-wise correlations. In addition to serving the overall statistical protocol, the graphical examination of pair-wise correlations may also serve as a starting point for meta-analysis and the synthesis of several published datasets. Of the statistical methods we present, plots of pair-wise correlations are best suited to meta-analysis. The other techniques (CPCA and group-specific correlation circles) require that the same indices be measured in all individuals or groups. This is a particular challenge in ecological immunology. Oftentimes, the number of measurements that can be taken from each individual is limited by the volume of blood that can be collected from a study subject of small body size. Moreover, many studies still employ different assays or at least different protocols to measure immune function.

**Figure 7:**
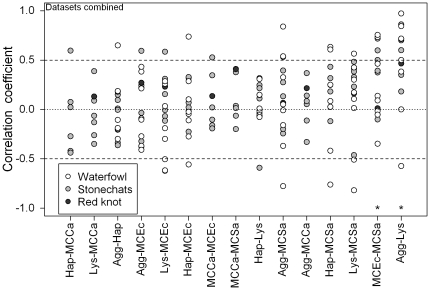
Dotplot showing correlation coefficients for pair-wise Pearson correlations between indices of immune function (see Table 1 for abbreviations) in an analysis combining waterfowl, stonechat and knot datasets. Each correlation coefficient for each species or subspecies is shown as a dot with its dataset of origin indicated in the shading. Waterfowl correlations are based on 5 to 8 individuals, stonechat correlations are based on 10 to 20 individuals, and knot correlations are based on 27 individuals. Asterisks indicate where weighted mean correlation coefficients [31] differed significantly from zero after sequential Bonferroni correction [30].

We illustrate the potential value and pitfalls of graphical examination of pair-wise correlations for meta-analysis by combining the three datasets presented in this paper (Figure 7). In this case, we plotted each correlation coefficient individually rather summarizing the overall variation with a boxplot. Datasets taken from the different studies were coded accordingly. The analysis confirmed that there was very little consistency in relationships among immune indices both among species (dots) and among datasets (shading). T-tests on mean correlation coefficients, weighted for sample size [31], demonstrated that only two relationships (hemagglutination and hemolysis, anti-*E. coli* and *S. aureus* capacities) differed significantly from zero; and even these relationships were not consistently positive (e.g. in the hemagglutination and hemolysis pair, one waterfowl species has a correlation coefficient of zero (SGPT) and another of -0.6 (ALCG)).

Inconsistencies in the relationships between immune indices identified by this exercise further emphasize immunological complexity. However, immunological interpretations are limited by other factors. Even this small group of datasets, which were collected by the authors over approximately a five-year period, lacked complete methodological uniformity. First, recall that microbicidal capacity was measured differently in waterfowl (plasma only) versus stonechats and knots (whole blood). Complicating matters further, methodology was confounded with taxonomy—similar species were measured similarly. This complication, however, reflects the reality of the broader ecological immunology community, in which research groups often focus on particular study species and customize immunological assays in one way or another. Second, recall that for the knot dataset we have multiple samples from each individual over an annual cycle. Because Buehler et al. [16] found significant variation among months we selected only measurements that were recorded during a quiescent winter period, which was most comparable to the other datasets. Intra-annual patterns demonstrate the importance of explicitly stating when in the annual cycle samples were collected, if independent studies are to be combined in meta-analyses.

Overall, the need for and utility of multivariate statistical methods is clear. Currently, methods like the protocol we describe are most useful and informative when applied within individual studies. Analytical integration among datasets remains a challenge; meeting this challenge will require judicious standardization of methodologies related to the collecting, processing and assaying of samples.

When considering the future of ecological immunology analyses, the potential importance of methods that can unite several multivariate datasets also comes to light. Additional datasets might, for example, include pathogen load variables, immunogenetic variables, and environmental (biotic and abiotic) variables. Thus, structural equation modelling (SEM) is a potentially powerful analytical method for ecological immunology. This method can be used to examine relationships between latent variables, which comprise multiple measured variables. A latent variable may itself be unmeasured (or even un-measurable; [23]). For example, multiple immune indices (i.e. the measured variables) could be used to estimate immune function (i.e. a latent variable) or several different “arms” of immune function (different latent variables for innate, acquired, etc.). Measures of pathogen richness and abundance could be similarly used to estimate pathogen pressure. SEM examines how each measured variable contributes to each latent variable (the measurement model) and how the latent variables relate to one another (the structural model). However, the field of ecological immunology will probably have to develop further before this method becomes a realistic option. In SEM, models must be specified *a priori*, but current limitations on our knowledge mean specifying a model for even a single latent variable, such as immune function, is unrealistic for most wild species. Nonetheless, once a stronger foundation has been established, the application of SEM might be an instructive research trajectory for ecological immunology.

## Supporting Information

Figure S1 Correlation circles for unrotated principal component analyses (PCA) on cellular indices of immune function among species of waterfowl (see Table 1 for abbreviations). Vectors are the loadings on PC1 (x-axis) and PC2 (y-axis). Vector length indicates the strength of the relationship and the angle between two vectors gives the degree of correlation (adjacent = highly correlated, orthogonal (90°) = uncorrelated, and opposite (180°) = negatively correlated). Shading indicates how indices of immune function were grouped in a previous varimax rotated PCA performed with all species combined [5]. Indices having the same shading were associated with the same PC in the previous analysis.
(TIF)

Figure S2 Correlation circles for unrotated principal component analyses (PCA) on indices of immune function (see Table 1 for abbreviations) measured in 27 individuals (a) and over 11 months (b) in red knots (*Calidris canutus*). Vectors are the loadings on PC1 (x-axis) and PC2 (y-axis). Vector length indicates the strength of the relationship and the angle between two vectors gives the degree of correlation (adjacent = highly correlated, orthogonal (90°) = uncorrelated, and opposite (180°) = negatively correlated). Shading indicates how the indices of immune function were grouped in a previous varimax rotated PCA performed with all individuals (a) or all months (b) combined [16]. Indices having the same shading were associated with the same PC in the previous analysis. Among individuals (a), monocytes correlated nearly equally across two PCs in the previous analysis [16], therefore it has darker grey shading with white lettering.
(TIF)

Table S1 Common principal components analysis (CPCA) of covariance matrices among waterfowl species for cellular indices of immune function. The table shows Flury's Decomposition of Chi Square using step-up and model building approaches (see Table 2 for details). Both methods indicate that covariance matrices among species share all PCs, but have differing eigenvalues (CPC). This CPCA is based on five matrices with six to eight observations per matrix because two species (NABD and MUSC) were excluded due to low levels of variability in one or more immune index.
(DOC)

Table S2 Mean correlation coefficients for pairwise Pearson correlations between plasma-based (a), and cellular (b), indices of immune function (see Table 1 for abbreviations) among species of waterfowl (n = 8 for plasma-based and n = 7 for cellular immune function). No mean correlation coefficients were significantly different from zero after sequential Bonferroni correction [30] (see text for statistical details).
(DOC)

Table S3 Mean correlation coefficients for pairwise Pearson correlations of indices of immune function (see Table 1 for abbreviations) among six stonechat subspecies. *P*-values indicating significant difference from zero after sequential Bonferroni correction [30] are bold (see text for statistical details).
(DOC)

Table S4 Mean correlation coefficients for pairwise Pearson correlations of indices of immune function (see Table 1 for abbreviations) among 27 individuals (a), and over 11 monthly measurements (b) in red knots (*Calidris canutus*). *P*-values indicating significant difference from zero after sequential Bonferroni correction [30] are bold (see text for statistical details).
(DOC)

Table S5 Loadings and eigenvalues for a varimax rotated principal component analysis (PCA) on indices of immune function measured in stonechat subspecies. The analysis was performed on data combined from six subspecies after statistically accounting for subspecies effects (see [5] for method).
(DOC)
