# Supplementary figures and images for: One Problem, Many Solutions: Simple Statistical Approaches Help Unravel the Complexity of the Immune System in an Ecological Context
Source: PLoS One. 2011 Apr 19;6(4):e18592. doi: 10.1371/journal.pone.0018592 (PMC3079723; doi:10.1371/journal.pone.0018592)

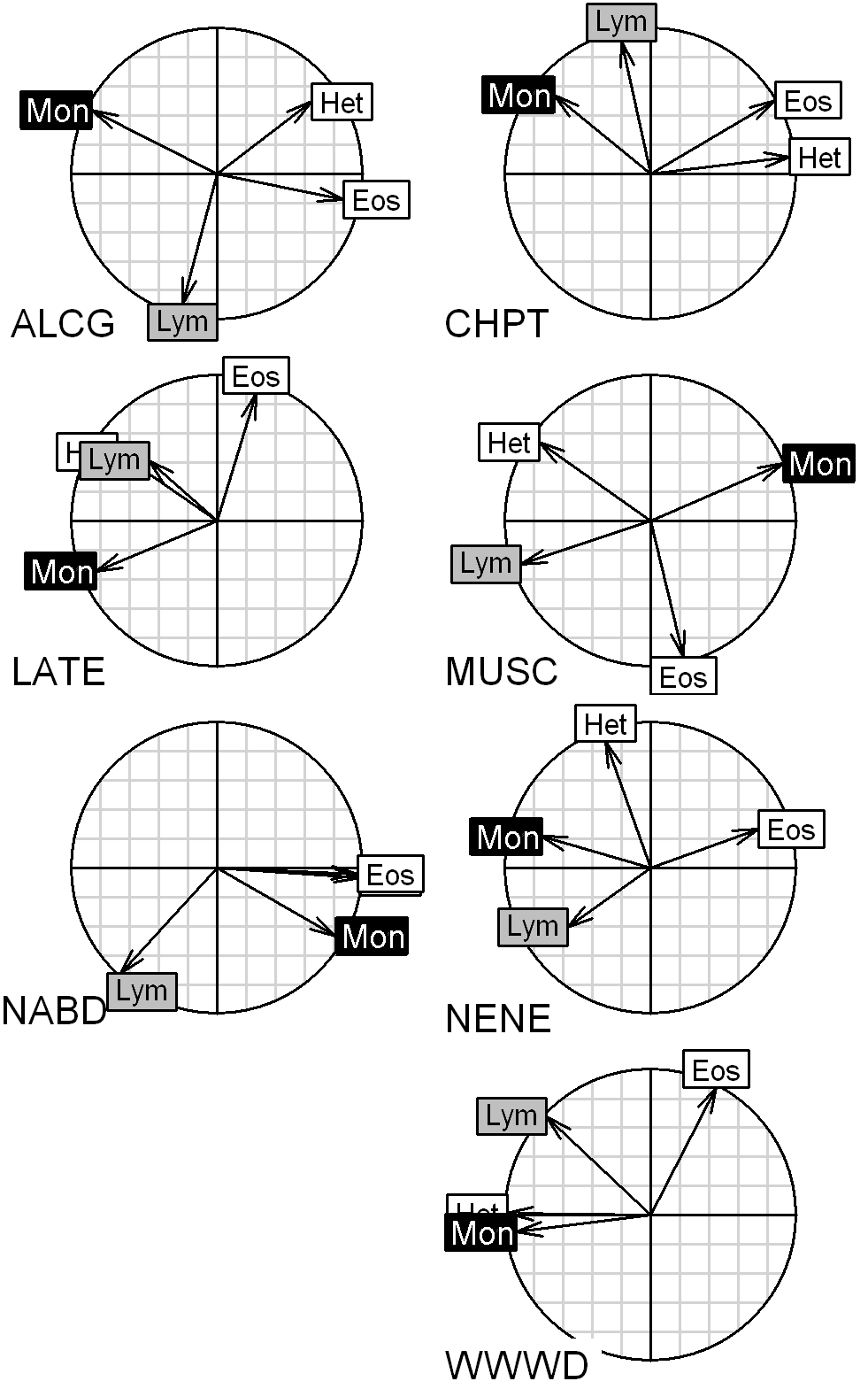

Supplement: Figure S1 — Correlation circles for unrotated principal component analyses (PCA) on cellular indices of immune function among species of waterfowl (see Table 1 for abbreviations). Vectors are the loadings on PC1 (x-axis) and PC2 (y-axis). Vector length indicates the strength of the relationship and the angle between two vectors gives the degree of correlation (adjacent = highly correlated, orthogonal (90°) = uncorrelated, and opposite (180°) = negatively correlated). Shading indicates how indices of immune function were grouped in a previous varimax rotated PCA performed with all species combined [5]. Indices having the same shading were associated with the same PC in the previous analysis. (TIF) [file pone.0018592.s001.tif]

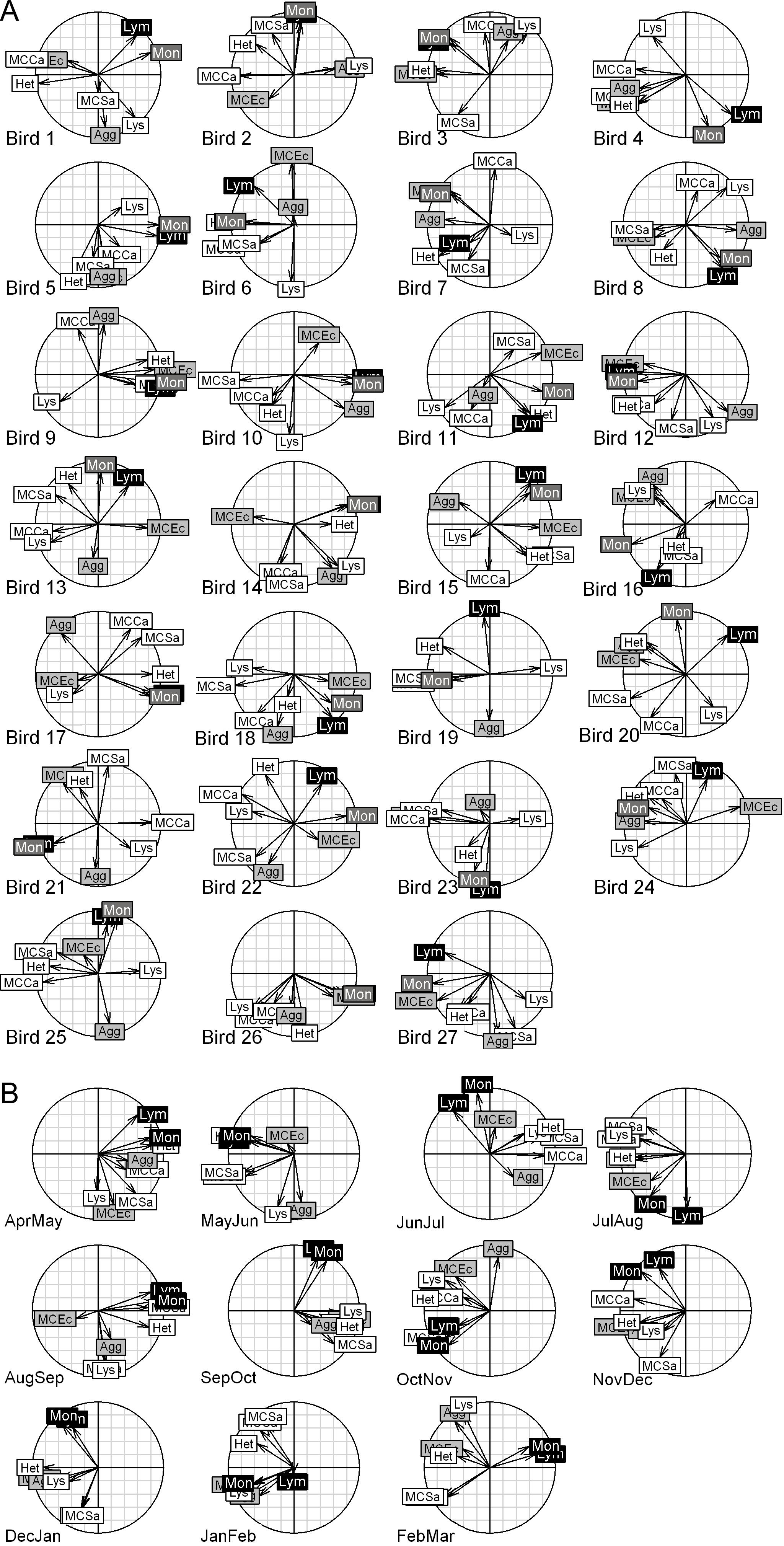

Supplement: Figure S2 — Correlation circles for unrotated principal component analyses (PCA) on indices of immune function (see Table 1 for abbreviations) measured in 27 individuals (a) and over 11 months (b) in red knots (Calidris canutus). Vectors are the loadings on PC1 (x-axis) and PC2 (y-axis). Vector length indicates the strength of the relationship and the angle between two vectors gives the degree of correlation (adjacent = highly correlated, orthogonal (90°) = uncorrelated, and opposite (180°) = negatively correlated). Shading indicates how the indices of immune function were grouped in a previous varimax rotated PCA performed with all individuals (a) or all months (b) combined [16]. Indices having the same shading were associated with the same PC in the previous analysis. Among individuals (a), monocytes correlated nearly equally across two PCs in the previous analysis [16], therefore it has darker grey shading with white lettering. (TIF) [file pone.0018592.s002.tif]
