# Supplementary material for: One Problem, Many Solutions: Simple Statistical Approaches Help Unravel the Complexity of the Immune System in an Ecological Context
Source: PLoS One. 2011 Apr 19;6(4):e18592. doi: 10.1371/journal.pone.0018592 (PMC3079723; doi:10.1371/journal.pone.0018592)
Supplement: Table S1 — Common principal components analysis (CPCA) of covariance matrices among waterfowl species for cellular indices of immune function. The table shows Flury's Decomposition of Chi Square using step-up and model building approaches (see Table 2 for details). Both methods indicate that covariance matrices among species share all PCs, but have differing eigenvalues (CPC). This CPCA is based on five matrices with six to eight observations per matrix because two species (NABD and MUSC) were excluded due to low levels of variability in one or more immune index. (DOC) [file pone.0018592.s003.doc]

Table S1

| Hierarchy | |  |  |  |  |  |
| --- | --- | --- | --- | --- | --- | --- |
| Higher | Lower |  | χ2 | df | *P* | AIC |
| Equality | Proport |  | 0.43 | 6 | 0.9986 | 102.5 |
| Proport | **CPC** |  | 64.23 | 18 | **<0.0001** | 114.0 |
| **CPC** | CPC(2) |  | 8.33 | 6 | 0.2152 | **85.8** |
| CPC(2) | CPC(1) |  | 16.27 | 12 | 0.1792 | 89.5 |
| CPC(1) | Unrelated |  | 13.21 | 18 | 0.7789 | 97.2 |
| Unrelated | --- |  |  |  |  | 120.0 |
