# Supplementary material for: One Problem, Many Solutions: Simple Statistical Approaches Help Unravel the Complexity of the Immune System in an Ecological Context
Source: PLoS One. 2011 Apr 19;6(4):e18592. doi: 10.1371/journal.pone.0018592 (PMC3079723; doi:10.1371/journal.pone.0018592)
Supplement: Table S2 — Mean correlation coefficients for pairwise Pearson correlations between plasma-based (a), and cellular (b), indices of immune function (see Table 1 for abbreviations) among species of waterfowl (n = 8 for plasma-based and n = 7 for cellular immune function). No mean correlation coefficients were significantly different from zero after sequential Bonferroni correction [30] (see text for statistical details). (DOC) [file pone.0018592.s004.doc]

Table S2

(a)

| **Variables** | **Mean** | **T** | **df** | ***P*** | **95% Confidence interval** |
| --- | --- | --- | --- | --- | --- |
| Lys-MCEc | -0.227 | -1.697 | 7 | 0.1336 | -0.503 to 0.091 |
| Lys-MCSa | -0.037 | -0.187 | 7 | 0.8568 | -0.469 to 0.409 |
| Agg-MCEc | -0.015 | -0.131 | 7 | 0.8992 | -0.279 to 0.251 |
| Lys-Hap | 0.038 | 0.809 | 7 | 0.4454 | -0.073 to 0.149 |
| Hap-MCEc | 0.040 | 0.261 | 7 | 0.8017 | -0.314 to 0.385 |
| MCSa-Agg | 0.041 | 0.193 | 7 | 0.8528 | -0.430 to 0.494 |
| Agg-Hap | 0.045 | 0.374 | 7 | 0.7196 | -0.237 to 0.321 |
| Hap-MCSa | 0.113 | 0.599 | 7 | 0.5682 | -0.324 to 0.511 |
| MCEc-MCSa | 0.280 | 1.729 | 7 | 0.1274 | -0.105 to 0.592 |
| Agg-Lys | 0.510 | 2.122 | 7 | 0.0715 | -0.064 to 0.831 |

(b)

| **Variables** | **Mean** | **t** | **df** | ***P*** | **95% Confidence interval** |
| --- | --- | --- | --- | --- | --- |
| Eos-Mon | -0.273 | -1.491 | 6 | 0.1866 | -0.629 to 0.178 |
| Eos-Lym | -0.043 | -0.392 | 6 | 0.7084 | -0.303 to 0.223 |
| Lym-Mon | 0.012 | 0.053 | 6 | 0.9593 | -0.480 to 0.497 |
| Het-Lym | 0.064 | 0.344 | 6 | 0.7426 | -0.373 to 0.478 |
| Het-Mon | 0.219 | 0.869 | 6 | 0.4184 | -0.383 to 0.690 |
| Eos-Het | 0.416 | 1.158 | 6 | 0.2907 | -0.456 to 0.881 |
