# Supplementary material for: One Problem, Many Solutions: Simple Statistical Approaches Help Unravel the Complexity of the Immune System in an Ecological Context
Source: PLoS One. 2011 Apr 19;6(4):e18592. doi: 10.1371/journal.pone.0018592 (PMC3079723; doi:10.1371/journal.pone.0018592)
Supplement: Table S3 — Mean correlation coefficients for pairwise Pearson correlations of indices of immune function (see Table 1 for abbreviations) among six stonechat subspecies. P-values indicating significant difference from zero after sequential Bonferroni correction [30] are bold (see text for statistical details). (DOC) [file pone.0018592.s005.doc]

Table S3

| **Variables** | **Mean** | **t** | **df** | ***P*** | **95% Confidence interval** |
| --- | --- | --- | --- | --- | --- |
| Hap-MCCa | -0.115 | -0.837 | 5 | 0.4407 | -0.439 to 0.235 |
| Lys-MCCa | -0.058 | -0.601 | 5 | 0.5742 | -0.296 to 0.187 |
| Agg-Hap | -0.040 | -0.536 | 5 | 0.6150 | -0.230 to 0.152 |
| Agg-MCCa | 0.038 | 0.448 | 5 | 0.6726 | -0.176 to 0.248 |
| Hap-Lys | 0.085 | 0.711 | 5 | 0.5092 | -0.219 to 0.374 |
| Hap-MCEc | 0.096 | 1.288 | 5 | 0.2543 | -0.096 to 0.282 |
| MCCa-MCEc | 0.101 | 0.893 | 5 | 0.4128 | -0.187 to 0.373 |
| Hap-MCSa | 0.114 | 1.345 | 5 | 0.2365 | -0.104 to 0.322 |
| Agg-MCEc | 0.118 | 0.886 | 5 | 0.4162 | -0.222 to 0.433 |
| MCCa-MCSa | 0.119 | 1.353 | 5 | 0.2341 | -0.107 to 0.333 |
| Lys-MCEc | 0.160 | 1.582 | 5 | 0.1744 | -0.101 to 0.400 |
| Agg-MCSa | 0.196 | 1.810 | 5 | 0.1301 | -0.083 to 0.446 |
| Lys-MCSa | 0.243 | 2.077 | 5 | 0.0924 | -0.059 to 0.503 |
| MCEc-MCSa | 0.396 | 3.395 | 5 | 0.0194 | 0.101 to 0.627 |
| Agg-Lys | 0.627 | 5.272 | 5 | **0.0033** | 0.360 to 0.798 |
