# Supplementary material for: One Problem, Many Solutions: Simple Statistical Approaches Help Unravel the Complexity of the Immune System in an Ecological Context
Source: PLoS One. 2011 Apr 19;6(4):e18592. doi: 10.1371/journal.pone.0018592 (PMC3079723; doi:10.1371/journal.pone.0018592)
Supplement: Table S4 — Mean correlation coefficients for pairwise Pearson correlations of indices of immune function (see Table 1 for abbreviations) among 27 individuals (a), and over 11 monthly measurements (b) in red knots (Calidris canutus). P-values indicating significant difference from zero after sequential Bonferroni correction [30] are bold (see text for statistical details). (DOC) [file pone.0018592.s006.doc]

Table S4

(a)

| **Variables** | **Mean** | **t** | **df** | ***P*** | **95% Confidence interval** |
| --- | --- | --- | --- | --- | --- |
| Lys-MCEc | -0.466 | -7.477 | 26 | **<0.0001** | -0.568 to -0.351 |
| Lym-MCCa | -0.177 | -2.950 | 26 | 0.0066 | -0.294 to -0.054 |
| Lym-MCSa | -0.179 | -2.130 | 26 | 0.0428 | -0.342 to -0.006 |
| Lym-Lys | -0.143 | -2.868 | 26 | 0.0081 | -0.242 to -0.041 |
| Het-Lys | -0.082 | -1.286 | 26 | 0.2096 | -0.211 to 0.049 |
| Lys-Mon | -0.080 | -1.409 | 26 | 0.1708 | -0.195 to 0.037 |
| Agg-Lym | -0.070 | -1.071 | 26 | 0.2939 | -0.202 to 0.065 |
| MCCa-Mon | -0.066 | -0.835 | 26 | 0.4112 | -0.225 to 0.096 |
| Lys-MCSa | -0.023 | -0.369 | 26 | 0.7152 | -0.152 to 0.106 |
| Agg-Mon | -0.003 | -0.052 | 26 | 0.9590 | -0.124 to 0.118 |
| MCSa-Mon | 0.007 | 0.105 | 26 | 0.9168 | -0.133 to 0.147 |
| Agg-MCSa | 0.046 | 0.556 | 26 | 0.5827 | -0.124 to 0.214 |
| Agg-Het | 0.046 | 0.735 | 26 | 0.4691 | -0.082 to 0.172 |
| MCCa-MCEc | 0.079 | 0.923 | 26 | 0.3646 | -0.096 to 0.249 |
| Agg-MCEc | 0.064 | 1.091 | 26 | 0.2852 | -0.056 to 0.182 |
| Lys-MCCa | 0.072 | 0.880 | 26 | 0.3869 | -0.095 to 0.234 |
| Agg-MCCa | 0.106 | 1.627 | 26 | 0.1157 | -0.028 to 0.236 |
| MCEc-MCSa | 0.129 | 1.666 | 26 | 0.1078 | -0.03 to 0.281 |
| Lym-MCEc | 0.129 | 2.301 | 26 | 0.0297 | 0.014 to 0.242 |
| Het-Lym | 0.192 | 2.904 | 26 | 0.0074 | 0.057 to 0.321 |
| MCEc-Mon | 0.226 | 3.104 | 26 | 0.0046 | 0.077 to 0.364 |
| Het-MCEc | 0.266 | 3.424 | 26 | **0.0021** | 0.109 to 0.411 |
| Het-Mon | 0.257 | 4.083 | 26 | **0.0004** | 0.130 to 0.376 |
| Agg-Lys | 0.273 | 3.912 | 26 | **0.0006** | 0.132 to 0.403 |
| Het-MCCa | 0.291 | 4.041 | 26 | **0.0004** | 0.146 to 0.424 |
| MCCa-MCSa | 0.320 | 4.035 | 26 | **0.0004** | 0.161 to 0.462 |
| Het-MCSa | 0.424 | 8.711 | 26 | **<0.0001** | 0.333 to 0.507 |
| Lym-Mon | 0.679 | 9.849 | 26 | **<0.0001** | 0.574 to 0.761 |

(b)

| **Variables** | **Mean** | **t** | **df** | ***P*** | **95% Confidence interval** |
| --- | --- | --- | --- | --- | --- |
| Lym-MCSa | -0.0709 | -1.023 | 10 | 0.3302 | -0.222 to 0.083 |
| Lym-MCEc | -0.0481 | -1.090 | 10 | 0.3013 | -0.146 to 0.050 |
| Lym-Lys | -0.0342 | -0.677 | 10 | 0.5136 | -0.146 to 0.078 |
| Lym-MCCa | -0.0154 | -0.198 | 10 | 0.8471 | -0.186 to 0.156 |
| Agg-Lym | -0.0076 | -0.169 | 10 | 0.8690 | -0.107 to 0.092 |
| Agg-Mon | 0.0188 | 0.264 | 10 | 0.7975 | -0.140 to 0.176 |
| MCCa-Mon | 0.0529 | 0.702 | 10 | 0.4984 | -0.115 to 0.217 |
| MCSa-Mon | 0.0808 | 1.061 | 10 | 0.3137 | -0.089 to 0.246 |
| Lys-Mon | 0.0987 | 1.898 | 10 | 0.0870 | -0.017 to 0.212 |
| Agg-MCSa | 0.1205 | 2.858 | 10 | 0.0170 | 0.027 to 0.212 |
| Agg-Het | 0.1331 | 1.777 | 10 | 0.1059 | -0.034 to 0.293 |
| MCEc-MCSa | 0.1359 | 1.740 | 10 | 0.1125 | -0.038 to 0.302 |
| MCCa-MCEc | 0.1405 | 2.156 | 10 | 0.0565 | -0.005 to 0.280 |
| Agg-MCEc | 0.1525 | 2.337 | 10 | 0.0415 | 0.007 to 0.292 |
| Lys-MCCa | 0.1712 | 2.859 | 10 | 0.0170 | 0.038 to 0.298 |
| Agg-MCCa | 0.1768 | 3.571 | 10 | 0.0051 | 0.067 to 0.282 |
| Lys-MCSa | 0.1834 | 3.197 | 10 | 0.0095 | 0.056 to 0.305 |
| Lys-MCEc | 0.1875 | 3.974 | 10 | 0.0026 | 0.083 to 0.288 |
| MCEc-Mon | 0.2076 | 3.815 | 10 | 0.0034 | 0.087 to 0.322 |
| Het-MCEc | 0.2143 | 3.908 | 10 | 0.0029 | 0.093 to 0.329 |
| Het-Lys | 0.2545 | 3.563 | 10 | 0.0052 | 0.097 to 0.399 |
| Het-Lym | 0.2614 | 3.180 | 10 | 0.0098 | 0.080 to 0.426 |
| Het-MCCa | 0.3198 | 6.520 | 10 | **0.0001** | 0.215 to 0.417 |
| MCCa-MCSa | 0.3199 | 4.453 | 10 | **0.0012** | 0.164 to 0.460 |
| Het-Mon | 0.3591 | 5.940 | 10 | **0.0001** | 0.231 to 0.475 |
| Agg-Lys | 0.3677 | 4.101 | 10 | **0.0021** | 0.174 to 0.534 |
| Het-MCSa | 0.4366 | 9.823 | 10 | **<0.0001** | 0.347 to 0.518 |
| Lym-Mon | 0.5419 | 6.989 | 10 | **<0.0001** | 0.391 to 0.664 |
