# Supplementary material for: One Problem, Many Solutions: Simple Statistical Approaches Help Unravel the Complexity of the Immune System in an Ecological Context
Source: PLoS One. 2011 Apr 19;6(4):e18592. doi: 10.1371/journal.pone.0018592 (PMC3079723; doi:10.1371/journal.pone.0018592)
Supplement: Table S5 — Loadings and eigenvalues for a varimax rotated principal component analysis (PCA) on indices of immune function measured in stonechat subspecies. The analysis was performed on data combined from six subspecies after statistically accounting for subspecies effects (see [5] for method). (DOC) [file pone.0018592.s007.doc]

Table S5

|  | PC1 | PC2 | PC3 |
| --- | --- | --- | --- |
| Hemagglutination (log2) | **0.83** | 0.15 | -0.16 |
| Hemolysis (log2) | **0.86** | 0.10 | 0.10 |
| Haptoglobin (mg/ml) | -0.13 | 0.20 | **0.86** |
| *E. coli* (prop. killed) | 0.12 | **0.78** | 0.00 |
| *S. aureus* (prop. killed) | 0.27 | **0.74** | 0.19 |
| *C. albicans*(prop. killed) | -0.24 | **0.55** | **-0.55** |
| **Eigenvalues** | 1.59 | 1.53 | 1.10 |
